# The importance of considering the evidence in the MTP 2014 Amendment debate in India – unsubstantiated arguments should not impede improved access to safe abortion

**DOI:** 10.3402/gha.v8.27512

**Published:** 2015-03-30

**Authors:** Mandira Paul, Kristina Gemzell Danielsson, Birgitta Essén, Marie Klingberg Allvin

**Affiliations:** 1 Department of Women's and Children's Health, Uppsala University, Akademiska Sjukhuset, Uppsala, Sweden; 2 Division of Obstetrics and Gynecology, Department of Women's and Children's Health, Karolinska Institutet, Karolinska University Hospital, Stockholm, Sweden; 3 School of Education, Health and Social Studies, Dalarna University, Falun, Sweden

**Keywords:** health policy, abortion, India, women's health

## Abstract

With the objective to improve access to safe abortion services in India, the Ministry of Health and Welfare, with approval of the Law Ministry, published draft amendments of the MTP Act on October 29, 2014. Instead of the expected support, the amendments created a heated debate within professional medical associations of India. In this commentary, we review the evidence in response to the current discourse with regard to the amendments. It would be unfortunate if unsubstantiated one-sided arguments would impede the intention of improving access to safe abortion care in India.

India has a progressive abortion law, the 1971 Medical Termination of Pregnancy (MTP) Act, amended last in 2002. Yet, judging from the number of maternal mortalities caused by unsafe abortions in India (9%), abortion services are not accessible to women (1). With the objective to improve access to safe abortion services, the Ministry of Health and Welfare with approval of the Law Ministry, published draft amendments of the MTP Act on October 29, 2014. This was to elicit opinions from the medical profession and the public before tabling the Act in Parliament. Instead of the expected support, the amendments created a heated debate within professional medical associations of India. Why are the amendments so controversial? Why this heated debate and what are the potential consequences for women? Can this debate lead to further delay of improving access to safe abortion for women in India? In this commentary, we review the evidence in response to the current discourse with regard to the amendments.

The MTP Act allows abortions up to 20 weeks gestation if the mother's life is at risk, if the fetus suffers severe abnormalities or, if the contraceptive method used fails to prevent pregnancy. Governmental facilities may provide abortions given that the medical practitioner is trained in abortion care. Private clinics can only provide abortion care after a governmental approval (2). In an effort to increase access to safe abortion services in India, the latest MTP amendments of 2002 and MTP rules of 2003 enabled decentralization of the certification system of private providers and facilities to district level and allowed for use of medical abortion pills outside of certified facilities, however, provided by certified providers (3). In spite of these efforts, the six states (Assam, Bihar, Madhya Pradesh, Odisha, Uttar Pradesh, and West Bengal) with highest incidence of abortions have the least amount of facilities per population, indication a persisting lack of access (3). Today, 12 years later, the MTP Act is due for another update. In summary, the MTP 2014 Amendment proposes to allow the following: non-physician practitioners as abortion care providers in the first trimester; abortions at a woman's request up to 12 weeks’ gestation; extend the period of legal abortion up to 24 weeks gestation for specific indications; and remove the requirement of two certified doctors for abortions after 12 weeks’ gestation (3). Upon release of these proposed amendments, key member associations of the Indian medical profession raised objections, mostly toward the proposal to broaden the provider base for early abortions (4–8). To impede progress of the amendment toward increased access to safe abortion care in India would be unfortunate, especially when considering the existing body of evidence on alternate providers in abortion care, both in India and in other low-, middle- as well as high-income countries (9).

India is the number one contributor to maternal deaths worldwide with its 50,000 annual maternal deaths, and the most recent figures suggest 9% of these to be due to unsafe abortion (1). In practice, this means that one woman dies due to unsafe abortion every 2 h, in a country in which abortion is legal. The major barrier to safe abortions in India is the lack of access to proper transport, poor road connectivity, lack of certified providers in remote areas, and non-availability of affordable services outside of the public health system, when it is unable to provide appropriate abortion services. (10, 11). This situation calls for evidence-based interventions to improve access to safe abortion services within the existing legal framework. Today, the MTP Act allows obstetricians and gynecologists or in-service trained MBBS doctors to conduct abortions (10). However, the in-service training opportunities for MBBS doctors and private practitioners are rare and the certification process for private clinics can take years. In addition, comprehensive abortion care is rarely included or discussed in basic education for MBBS doctors (10). Inadequate basic and in-service training in combination with slow administration of private provider certification undermines women's ability to access legal and safe abortion services – a violation of women's reproductive and human rights. As a measure to improve this situation, the 2014 Amendments suggest a broadening of the provider base for early abortions. The amendments propose to, in addition to medical doctors, allow registered healthcare providers defined as qualified Ayurvedic, Unani, Siddha, and Homeopathy (AYUSH) practitioners; nurses; and auxiliary nurses–midwives to provide early abortion, after specified training. Studies from India suggest an interest from alternate providers to be trained in MTP and to be allowed to provide early abortions (12), in contrast to the lack of interest seen among MBBS doctors (13).

Several Indian studies, both theoretical and empirical, suggest the feasibility and benefits of allowing alternate providers in early abortion care in India. However, the empirical evidence refers to nurses and Ayurvedic doctors specifically (10–12, 14–16). Other studies from similar contexts carried out in countries such as Nepal, Bangladesh, Vietnam, and Cambodia prove feasibility of alternate providers of safe abortion, especially when they use medical methods of abortion (mifepristone and misoprostol) (17–26). In the ongoing MTP amendment discourse in India, one publication has achieved extra attention, namely the Indian study confirming the equivalence of Ayurvedic doctors or nurses with fresh MBBS doctors in terms of assessment of gestational length, eligibility for medical abortion, and abortion outcome. Importantly, the study emphasizes the importance of sufficient and quality training before allowing providers to provide medical abortion services. In the study, the study providers went through a 10-day, high-quality standard abortion training before the initiation of the study. Subsequently, during the study, a skilled abortion provider, referred to as the ‘verifier’, prescribed the medical abortion pills and verified the study providers’ assessments (11). Although the study attracted criticism and questioning, it was published in 2011 in a peer-reviewed journal by experienced researchers. Hence, the arguments stating that enabling alternate providers in abortion care would jeopardize women's lives, currently voiced by many of those opposing the amendments, are based on unsubstantiated grounds.

Arguments articulated, by those opposing the amendments, are the concerns of the many women that currently have to seek care for post-abortion complications after being provided with poor-quality abortion pills or instructions by some untrained provider, pharmacist, or ‘quack’ in the village. Based on this experience, those opposing the amendments argue that allowing alternate providers to provide abortions would result in more women seeking post-abortion care for complications and consequently more maternal mortality and morbidity. However, having a trained provider in abortion services cannot be compared with having an untrained provider, pharmacist or ‘quack’ providing abortion services in the village. The amendments clearly state that providers need to be trained before providing abortion services. To enable task sharing in abortion care can increase access to safe abortions, especially in rural settings. Moreover, medical methods of abortion mimics the process of miscarriage, a situation that can occur naturally and rarely requires specific care (27). However, having a trained provider in abortion care can increase safety and efficacy of the method.

We already know that women in need of an abortion will obtain one whether safe or unsafe (28). We also know that abortion services can be simplified, emphasizing the decreased roll of the provider in abortion care. For example, women can carry out abortion supported over telemedicine (29, 30). Women can both safely administer misoprostol at home (31, 32), and effectively assess their abortion outcome with a low-sensitivity pregnancy test at 2 weeks (33, 34) given that they are provided with appropriate counseling (32, 35). Given these advances in abortion care, the move to liberalize access through alternate providers suggested in the 2014 amendment is a step in the right direction. Whether the task sharing should include both surgical and medical methods of abortion may still be debated; however; there is sufficient evidence of the feasibility of task sharing in the provision of medical methods of abortion. With this we would like to reason that allowing alternate providers to be trained to assess women's eligibility for abortion and subsequent provision of safe medical abortion would decrease the need for post-abortion care and would increase women's access to safe, acceptable and affordable abortion services in their setting. Moreover, providing options of simplified medical abortion should be implemented already from the start, allowing the woman an active role in the abortion care and decreasing the workload of the health care providers.

We do not claim that the proposed amendments are flawless, nor do we claim that some of the concerns raised in relation to the amendments should not be taken into consideration. In addition, it will be important to assess the proposed MTP Act and accompanying rules (once released) together, to better understand the context in which the Act will be implemented. However, we do aim to elucidate the existing evidence contrasting the acclaimed arguments proposing the lack of safety with alternate providers of early abortion. It is time that the public health system of India moves from the discredited ‘dilatation & curettage’ or ‘D&C’ to safer and modern methods of abortion such as medical abortion and vacuum aspiration (33). These methods can easily be implemented at a primary health care level, provided by alternate providers and hence increase access to safe abortion services (10, 15) and respect women's reproductive rights. However, for task sharing to be successful, the importance of accurate and quality training and guidelines must be emphasized. Moreover, such training needs to be made available throughout India, and in combination with monitoring and evaluation to ensure quality and safety it can successfully expand the provider base and increase access to safe abortion services.
